# Spontaneous Rupture of a Giant Hepatic Hemangioma – Sequential Management with Transcatheter Arterial Embolization and Resection

**DOI:** 10.4103/1319-3767.61240

**Published:** 2010-04

**Authors:** Vaibhav Jain, Vijay Ramachandran, Rachana Garg, Sujoy Pal, Shivanand R. Gamanagatti, Deep N. Srivastava

**Affiliations:** Department of Radiology, All India Institute of Medical Sciences, Delhi, India; 1 Department Gastro-intestinal Surgery, All India Institute of Medical Sciences, Delhi, India

**Keywords:** Hemoperitoneum, giant hepatic hemangioma, liver tumor, transcatheter arterial embolization

## Abstract

Hemangioma is the most common benign tumor of liver and is often asymptomatic. Spontaneous rupture is rare but has a catastrophic outcome if not promptly managed. Emergent hepatic resection has been the treatment of choice but has high operative mortality. Preoperative transcatheter arterial embolization (TAE) can significantly improve outcome in such patients. We report a case of spontaneous rupture of giant hepatic hemangioma that presented with abdominal pain and shock due to hemoperitoneum. Patient was successfully managed by TAE, followed by tumor resection. TAE is an effective procedure in symptomatic hemangiomas, and should be considered in such high risk patients prior to surgery.

Liver hemangiomas have prevalence between 0.4–7.4% in autopsy studies.[1] One of the uncommon (1–4%) but often fatal complications is rupture of the tumor with hemoperitoneum, which has a high mortality rate ranging from 60–75%.[1,2] In a recent review of literature, the operative mortality rate of ruptured hemangioma was reported to be 36.4%.[3] Preoperative transcatheter arterial embolization (TAE) has shown excellent results with no operative mortality in four patients with spontaneous rupture of liver hemangioma reported so far in the literature.[3] We report a case of giant hemangioma of the liver who presented with abdominal pain and shock due to hemoperitoneum as a result of spontaneous rupture; that was managed successfully with TAE followed by tumor resection.

## CASE REPORT

**Figure 1:**
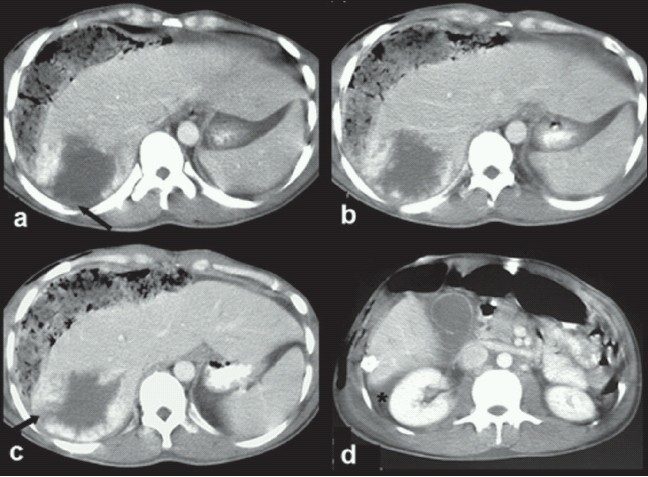
Axial CECT images of liver (a–d) show a mass in segments 6 and 7 with peripheral nodular enhancement suggestive of a hemangioma. Note a rent on the postero-superior aspect of the tumor (arrows), indicating focal rupture. High density peritoneal fluid is seen in perihepatic region (asterisk)

**Figure 2:**
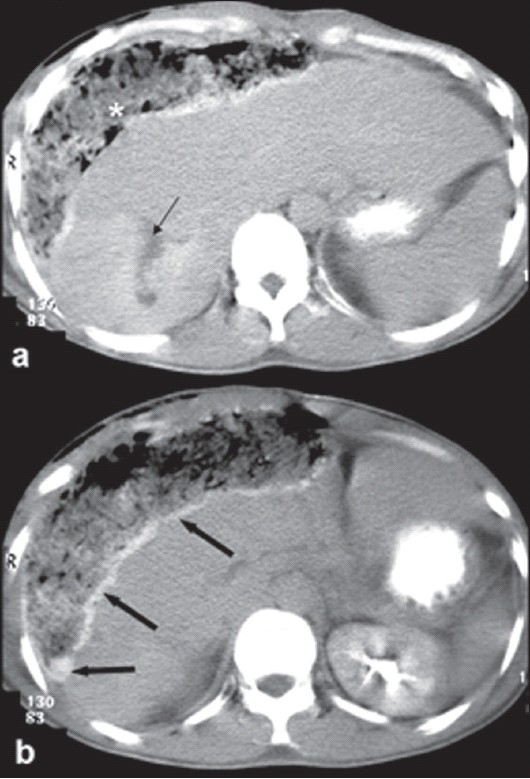
Delayed CT image (a) shows progressive centripetal filling-in of the tumor with contrast, confirming the diagnosis of a hemangioma, with a central non-enhancing area (arrow) suggestive of a fibrotic scar. Pack of sponges from the previous surgery (asterisk) can be seen at the liver surface. In addition, hyperdensity seen along the liver surface (solid arrows-b) represents active contrast extravasation

A 31-year-old man experienced sudden onset of abdominal pain and tenderness in the right hypochondrium. There was no history of trauma, and no history of significant disease in the past. He underwent laparotomy at a primary health care centre, owing to a misdiagnosis of ruptured liver abscess with peritonitis. On exploration, hemoperitoneum with active bleeding from the liver surface was found. Due to lack of diagnosis of the possible cause of bleeding, perihepatic packing was done with sponges, and the patient was referred to our tertiary care institute. The patient received three units of packed cells prior to shifting to our center. On arrival, patient was pale and in hemorrhagic shock. The blood pressure was 76/50 mmHg, pulse was 124 beats per min and feeble. Abdomen was distended and diffusely tender. Laboratory investigation revealed hemoglobin of 6.8 g/dl, hematocrit 26%, and high transaminase levels. After initial resuscitation with intravenous fluids and packed red blood cells (RBC), the patient underwent a contrast enhanced computerized tomography (CECT) scan on a spiral computerized tomography (CT) scanner. CT scan demonstrated a peripherally enhancing lesion in right lobe of liver, located in segments VI and VII [Figure 1]. The largest dimension of the lesion was 11 cm in the craniocaudal direction, and transverse and antero-posterior measurements were 9.7 and 7.3 cm respectively. The site of rupture was clearly seen as a rent in the enhancing margin of the lesion at the postero-superior aspect [Figure 1 – arrows]. On delayed scans, the lesion showed characteristic centripetal filling-in of contrast, typical of a hepatic hemangioma, with a small central non-enhancing fibrous scar [Figure 2a]. Delayed images also revealed hyperdensity tracking along the liver surface and settling in the dependent part [Figure 2b], indicating the presence of active contrast extravasation. There were associated hemoperitoneum and mild bilateral pleural effusions. Remnant liver parenchyma was normal with no other focal lesion. The sponges from previous laparotomy were also identified. Based on CT scan, a diagnosis of giant hepatic hemangioma with rupture and hemoperitoneum was made and patient was shifted to angiography suite within 12 hours of arrival at our center after adequate resuscitation. The patient was normotensive but tachycardic at the time of shifting to the angiography suite.

**Figure 3:**
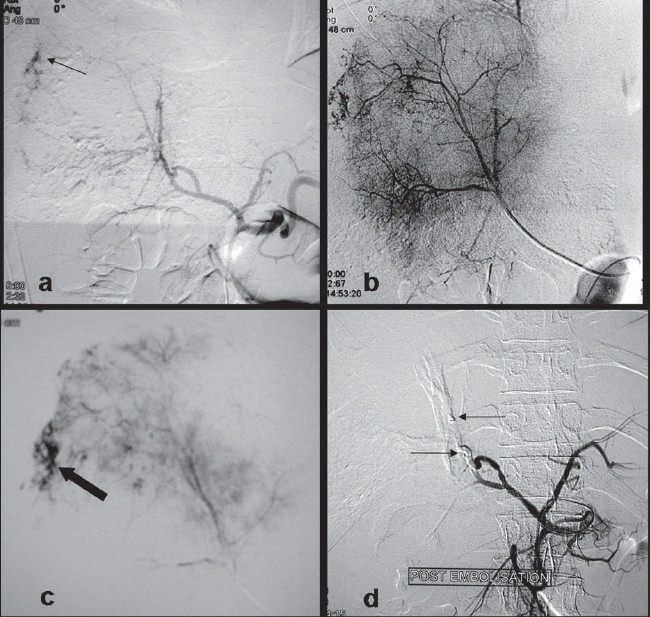
(a) Celiac angiogram showing extravasation of contrast from the region of right lobe of liver (arrow). Selective angiogram of right hepatic artery, immediate (b) and delayed (c) images, show characteristic pooling and puddling of contrast in the liver hemangioma as well as the active arterial extravasation (solid arrow). (d) Post embolization angiogram confirms lack of any active contrast extravasation. The steel coils can be seen as filling defects within the principal feeding arteries (arrows).

Digital Subtraction Angiography (DSA) revealed typical angiographic findings of a liver hemangioma with pooling and puddling of contrast in the right lobe lesion. Arterial contrast extravasation was noted on the celiac angiogram, after which a selective right hepatic arteriogram was done that confirmed active contrast extravasation from the hemangioma [Figure 3].

The hemangioma was embolized in a single session, first using PVA particles for embolisation of tumor interstices, and then the principal arteries were occluded using two steel coils [Figure 3]. PVA particles 500–750 microns (Cook Bloomington Inc U.S.A) were used for embolization and were delivered using a Cobra catheter. Deep catheterization obviated the need for using a microcatheter. The vitals of patient immediately stabilized, and a check angiogram showed successful embolisation with no contrast leak.

**Figure 4:**
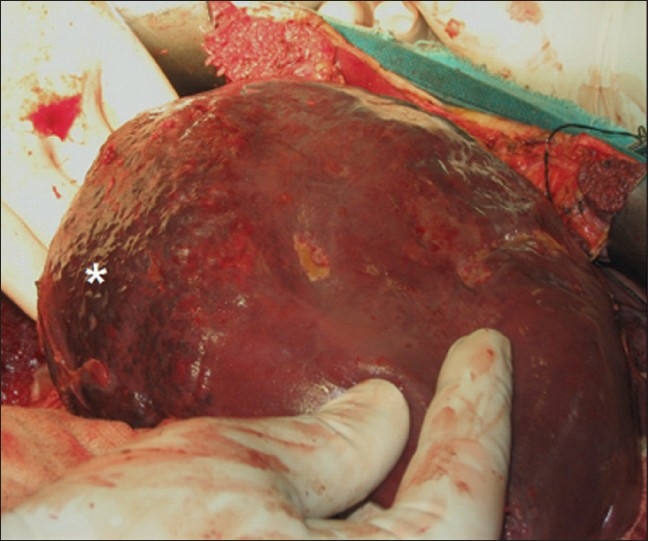
Peroperative photograph showing the mobilized liver with the hemangioma seen involving the segments VI and VII (asterisk)

The patient received a total of six transfusions perioperatively. When the general condition of the patient stabilized, he underwent laparotomy for removal of surgical packs. After pack removal, surgeons did not note any active bleed, and a large hemangioma involving segments VI and VII, which was ruptured on the posterior surface was noted [Figure 4]. The hemangioma could be completely enucleated without significant blood loss. Histological examination revealed a cavernous hemangioma of 11.5×10 cm diameter. The postoperative course was largely uneventful except for mild fever and right pleural effusion which necessitated tube thoracostomy. The patient fully recovered and discharged three weeks following surgery. Patient was asymptomatic on follow-up at two months.

## DISCUSSION

Cavernous hemangiomas are the most common benign neoplasm of liver and are often incidentally detected during abdominal imaging done for unrelated clinical indications.[4] Most of them are small and asymptomatic and do not require any intervention. Giant hemangiomas are the ones that are larger than 4 cm in diameter.[5] Absolute surgical indications for hepatic hemangioma are spontaneous or traumatic rupture with hemoperitoneum, intratumoral bleeding, and consumptive coagulopathy (Kasabach-Merritt syndrome). Persistent abdominal pain, obstructive jaundice, portal hypertension, superficial location of tumors larger than five cm with a risk of trauma, and an uncertain diagnosis are relative surgical indications.[6,7] Rupture of hemangioma with hemoperitoneum, as in our case, is the most dreaded complication and is often fatal if not promptly managed.[1–3] The first case of spontaneous rupture of a hepatic hemangioma was described by Van Haefen in 1898 in an autopsy case.[8] In 1961, Swell and Weiss[9] reviewed 12 cases of spontaneous rupture of hemangiomas from literature and reported the mortality rate to be as high as 75%.

Recent studies have emphasized the role of TAE in the effective treatment of symptomatic hemangiomas, progressively growing hemangiomas and those at risk of bleeding.[10,11] However, the use of TAE as an alternative to surgery in the management of ruptured liver tumors is controversial because of the fear of causing ischemia, intracavitary bleeding or infection.[12] Successful use of TAE before surgical resection of ruptured hepatic hemangioma was first reported by Yamamoto *et al* in 1991.[13] Since then, three more such cases have been reported in the literature, with no patient mortality.[3,14,15] The present case adds to the list of such patients.

In our case, the lesion was subcapsular and located in segment VII of right lobe. The likelihood of spontaneous ruptures is unknown but large, subcapsular lesions are considered to be at greater risk.[12] Abdominal CECT not only established the diagnosis of ruptured hemangioma but also demonstrated the site of rupture and suggested the possibility of active intra-abdominal bleeding which was confirmed on angiography. Perihepatic packing done prior to referral may have contributed to reduction in the rate of bleeding, thus facilitating angiography. The choice of embolic material depends upon the initial angiographic appearance and the experience of the radiologist. Various materials like gelfoam, polyvinyl alcohol (PVA) particles, steel coils, as well as isobutyl cyanoacrylate have been used.[10] We used PVA particles for initial embolization of vascular interstices and the principal arteries were embolized using steel coils, without any significant complication.

Tumor resection after TAE resulted in minimal per-operative blood loss in our patient. In cases of rupture, TAE results in stanching or reducing the hemorrhage, thereby improving the general condition of patient, thus making subsequent hepatic resection a safer procedure.[5] Suzuki *et al.* observed a significant improvement in coagulative factors and a decrease in intraoperative blood loss, in patients with consumptive coagulopathy related to intravascular coagulation in hemangioma, that were treated with preoperative TAE.[16]

To conclude, in a patient presenting with acute abdominal pain, with hitherto no known abdominal problem, spontaneous rupture of a hepatic tumor like hemangioma should also be kept as a rare differential. In addition, our case substantiates the limited available literature regarding validity of preoperative transcatheter arterial embolization and its usefulness in significantly improving the outcome in patients undergoing surgery for a ruptured hepatic hemangioma.
